# Low skeletal muscle mass as an early sign in children with fabry disease

**DOI:** 10.1186/s13023-023-02806-2

**Published:** 2023-07-21

**Authors:** Zhihong Lu, Guoping Huang, Ling Yu, Yan Wang, Langping Gao, Li Lin, Lidan Hu, Jianhua Mao

**Affiliations:** 1 Department of Nephrology, Children’s Hospital, Zhejiang University School of Medicine, National Clinical Research Center for Child Health, National Children’s Regional Medical Center, Hangzhou, China; 2 Children’s Hospital, Zhejiang University School of Medicine, National Clinical Research Center for Child Health, National Children’s Regional Medical Center, Hangzhou, China

**Keywords:** Fabry disease, Children, Skeletal muscle, Absorptiometry

## Abstract

**Background & aims:**

Fabry disease (FD) is a rare X-linked metabolic storage disorder due to the deficiency of lysosomal α-galactosidase A which causes the accumulation of glycosphingolipids throughout the body. Underweight and low BMI have been occasionally reported in FD patients previously. Whether underweight is common in the early stage of FD and body composition analysis to determine the cause have not been reported.

**Methods:**

Children who were diagnosed with FD in the Children’s Hospital of Zhejiang University School of Medicine from July 2014 to December 2022 were enrolled. Clinical data were obtained from medical records. Whole body dual energy X-ray absorptiometry scans (DXA) were used to assess body composition (fat mass, FM; fat free mass, FFM and bone mass) according to the International Society of Clinical Densitometry’s standard operating method. Whole body muscle mass was calculated as fat-free mass minus bone mass. Appendicular skeletal muscle mass (ASM) was calculated as the sum of the arm and the leg muscle mass. The FM, FFM, ULSM and LLSM indices were calculated by dividing the total FM, FFM, and upper and lower limb skeletal muscle mass (ULSM and LLSM) by the height squared.

**Results:**

A total of eighteen children (14 boys and 4 girls) were enrolled. Thirteen boys had the classical phenotype, and five children (1 boy with the N215S mutation and 4 girls) had the late-onset phenotype. Seven children with the classical phenotype (53.8%) and two of the five children (40%) with the late-onset phenotype had abnormal BMIs. Sixteen of the eighteen children (88.9%) had a height in the normal range, suggesting that low BMI was mainly due to underweight. By DXA body composition analysis, the FMI was abnormal in 3 children (2 boys and 1 girl), and the FFMI was abnormal in 12 children (9 boys and 3 girls). For the classical phenotype, 2 of the 13 children (15.4%) had abnormal FMI values, while 10 (76.9%) had abnormal FFMI values. Eight patients (61.5%) with the classical phenotype had a significant reduction in muscle mass index, ASM index and LLSM index values compared with age- and sex- matched Chinese controls. Late-onset patients also had mild low skeletal muscle mass compared to controls. The results suggested that low skeletal muscle mass is common in early FD.

**Conclusions:**

This is the first study to examine body composition and muscle mass in early Fabry disease patients. Low skeletal muscle mass is a common early symptom in children with Fabry disease, suggesting that skeletal muscle is significantly affected in the early stages of FD.

**Supplementary Information:**

The online version contains supplementary material available at 10.1186/s13023-023-02806-2.

## Introduction

Fabry disease (FD) is a rare X-linked storage disorder resulting from deficient activity of lysosomal hydrolase α-galactosidase A (α-Gal A) caused by genetic defects in the *GLA* gene [1]. This α-Gal A deficiency causes the accumulation of globotriaosylceramide (GL-3 or GB3) and related glycosphingolipids (Lyso-GL-3) in many cell types. The predominant pathological accumulation of GL-3, involves vascular endothelial cells and other cell types, such as neural cells, cardiomyocytes, and renal podocytes, leading to cellular dysfunction, tissue remodelling, fibrosis, ischaemia and, ultimately severe end-organ damage [2]. The incidence ranges between 1:40,000 and 1:117,000 in live born males worldwide [3, 4].

Early manifestations of patients with typical FD include neuropathic limb pain, hypohidrosis, angiokeratoma, corneal opacity, and gastrointestinal discomfort. Gastrointestinal Involvement is very common in FD, affecting up to 50% of female patients and almost 60% of untreated children [5]. Underweight and low body mass index (BMI) have been occasionally reported in FD patients [6], which was previously thought to mainly be related to gastrointestinal tract involvement. Whether underweight is common in the early stage of FD and body composition (BC) analysis to determine the cause have not been reported. In the present study, clinical data were collected from 18 children diagnosed with FD in the Children’s Hospital of Zhejiang University School of Medicine from July 2014 to December 2022. BMI and BC, including appendicular skeletal muscle mass (ASM) and related indices, were calculated in these children.

## Materials and methods

### Patients

A total of 18 children who were hospitalized in the Department of Nephrology, the Children’s Hospital of Zhejiang University School of Medicine (Hangzhou, China) from July 2014 to December 2022 were enrolled. They came from 6 provinces and 13 regions in China and were referred from other hospitals for the diagnosis of FD. All the clinical data collected came from tests done by children at the time of their FD diagnosis. Each patient’s family gave written informed consent for the collection of clinical data (2020-IRB-187-A1).

All the children were confirmed to have FD by enzyme activity, biomarker, and genetic tests. GLA variants are listed using transcript NM_000169.2. An enzymatic assay on dried blood spots collected on filter paper was used for screening [7]. The pathogenicity of the variant was verified according to ACMG criteria [8].

### Clinical evaluations

**Fig. 1:**
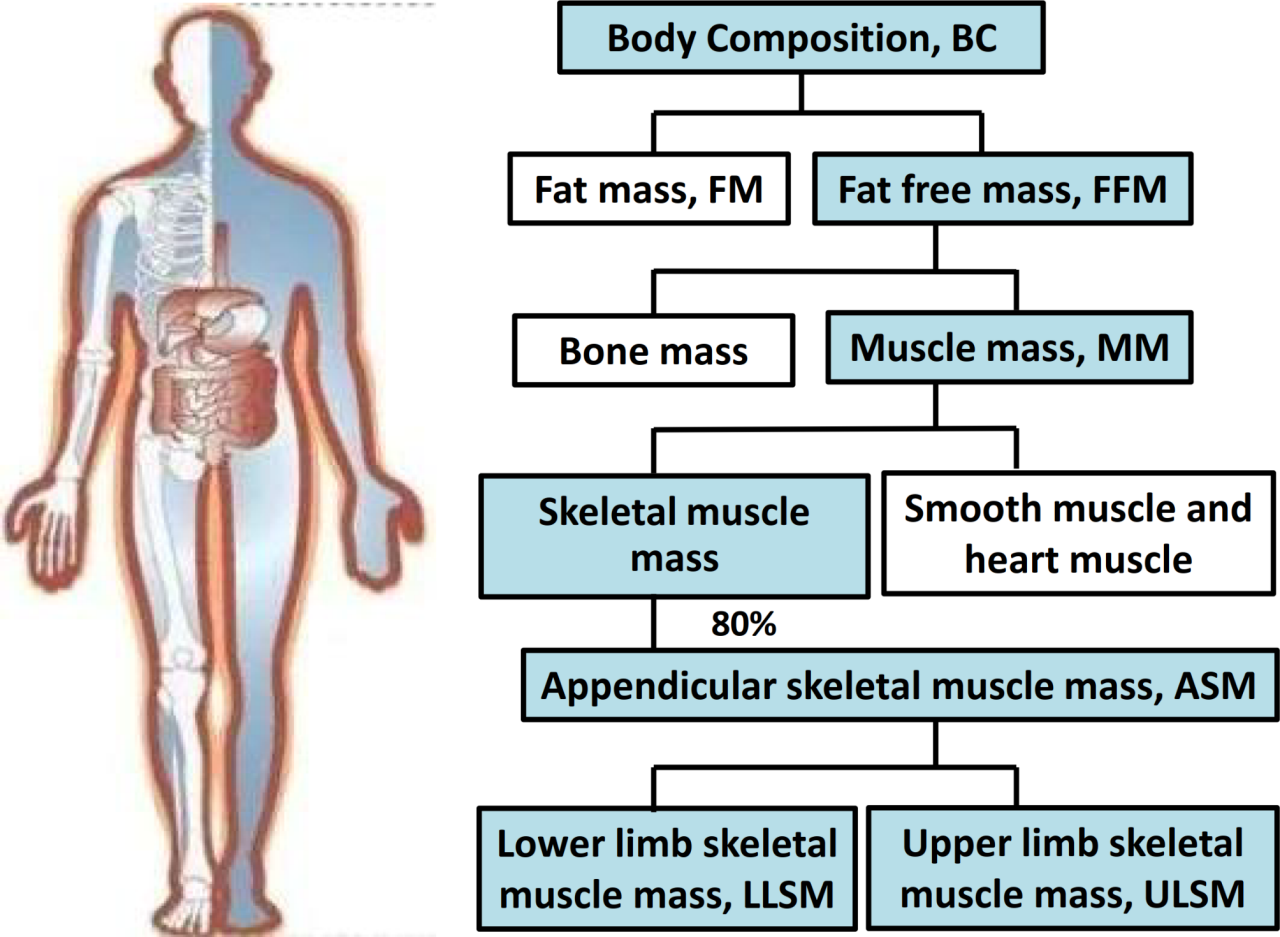
Whole body DXA scan analysis of body composition

Clinical data such as age, sex, liver and kidney function, and prealbumin, albumin, haemoglobin and calcium levels were obtained from medical records. Whole body dual energy X-ray absorptiometry (DXA) scans (Hologic Discovery-WI/CI, Bedford, MA, USA) were used to assess BC (fat mass, fat free mass and bone mass) according to the International Society of Clinical Densitometry’s standard operating method. Height and weight were measured at the time of the DXA scan. The classification of body composition indices is shown in Fig. 1. BMI was calculated as weight in kilograms divided by height in metres squared. Whole body muscle mass was calculated as fat free mass minus bone mass. Appendicular skeletal muscle mass (ASM) was calculated as the sum of the arm and the leg muscle mass (MM). The fat mass index (FMI; kg/m^2^), fat-free mass index (FFMI; kg/m^2^), upper limb skeletal muscle mass index (ULSMI; kg/m^2^) and lower limb skeletal muscle mass index (LLSMI; kg/m^2^) were calculated by dividing the total fat mass (FM), fat-free mass (FFM), upper limb skeletal muscle mass (ULSM) and lower limb skeletal muscle mass (LLSM) by the height squared.

### Statistical analysis

Statistical analyses were performed with SPSS software (version 17.0, SPSS). The results are expressed as the mean ±standard deviation. Standard Z score conversion was carried out for the body composition index, and the 95% confidence interval was calculated. The paired sample T test was used to compare the body composition index of children with FD with that of healthy children. Pearson correlation analysis was used to analyse the correlation between the ASMI and the indicators. The significance level for all statistical tests was set to α = 0.05.

## Results

Thirteen boys with the classical phenotype and five children (one boy with N215S mutation and 4 girls) with the late-onset phenotype were enrolled in the study. The clinical characteristics of the eighteen children (14 boys and 4 girls, mean age 11.6±2.7 years, age range: 6–17 years) are shown in Table 1. None of the children had significant long-term symptoms of nausea, vomiting, abdominal distension or pain, except 5 patients who had intermittent diarrhoea alternating with constipation. Liver and kidney function and prealbumin, albumin, and haemoglobin levels in blood were normal in all children. Two children had mild low blood calcium levels, and eleven had low 25-hydroxyvitamin D levels. No children had received specific treatment for FD, such as enzyme replacement therapy.

**Table 1:** Clinical characteristics of pediatric fabry patients

Case No. (n = 18)	Age	Gendar	cDNA Variation	Protein Variation	DBS AGAL activity (µmol/L/h)	DBS Lyso-GL-3 (ng/ml)	Birth Wt(Kg)	GI symptoms	ALT (U/L)	Cr (µmol/L)	Albumin (g/L)	PAB (mg/L)	Hemoglobin(g/L)	CK (U/L)	Calcium (mmol/L)	25(OH)D (nmol/L)
1^Δ^	14Y	M	c.424T > C	C142R	0.32	80.45	3.75	Y	11	85	42.4	177.8	112	66	2.23	32.3
2^#^	16Y9M	F	c.335G > A	R112H	2.1	0.55	4.25	N	8	73	36	241.3	115	49	2.12	29
3^#^	6Y	F	c.335G > A	R112H	1.31	0.51	2.4	N	10	51	37	161.9	131	88	2.16	33.2
4^#^	11Y10M	M	c.644 A > G	N215S	0.6	7.01	3.15	N	18	51	44.4	258.5	147	108	2.46	37.8
5^Δ^	11Y5M	M	c.140G > A	Trp47*	0.4	92.98	3.35	N	5	35	44.5	204.6	114	78	2.24	25.8
6^Δ^	13Y6M	M	c.3G > A	Met1?	0.34	74.02	3.5	Y	19	31	41.5	257.7	127	85	2.23	43.4
7^Δ^	9Y9M	M	c.100 A > G	N34H	0.33	64.05	3.8	N	16	37	43.2	216	120	67	2.42	42.9
8^Δ^	9Y9M	M	c.100 A > G	N34H	0.4	65.03	3.5	N	12	46	43.8	215.2	126	87	2.41	37.8
9^#^	11Y11M	F	c.100 A > G	N34H	1.64	2.77	3.0	N	10	38	46.2	259.6	142	120	2.41	22.9
10^Δ^	8Y1M	M	c.776 C > T	P259L	0.35	75.16	3.15	N	10	30	44.4	189.9	119	86	2.39	27.1
11^Δ^	12Y9M	M	c.334 C > T	R112C	0.35	59.73	3.8	N	10	46	43.9	222.4	123	95	2.31	17.7
12^Δ^	10Y10M	M	c.334 C > T	R112C	0.45	69.44	3.4	N	26	34	39.9	202.1	110	85	2.3	29.2
13^Δ^	8Y2M	M	c.782G > T	G261V	0.28	71.33	3.15	N	13	34	44.4	218.4	120	51	2.42	28.1
14^#^	7Y11M	F	c.782G > T	G261V	1.6	4.52	3.1	N	11	38	46.0	217.6	136	109	2.43	35.8
15^Δ^	12Y9M	M	c.72G > A	Trp24*	0.32	115.63	3.3	N	9	56	41.6	227.9	124	94	2.31	24.4
16^Δ^	13Y9M	M	C.486 G > A	Trp162*	0.67	72.6	3.8	N	8	54	42	238.8	134	100	2.45	35
17^Δ^	17Y	M	c.1072_1074delGAG	Glu358del	0.26	65.57	3.55	Y	14	64	42.6	165.9	141	44	2.35	28.5
18^Δ^	8Y6M	M	c.454_456dupTAC	Tyr152dup	0.86	66.96	3.2	N	20	38	46.1	205.1	133	93	2.50	44.7

Δ Classical Fabry disease; # Late-onset Fabry disease. References to clinical classification of FD patients: Arends M, Wanner C, Hughes D,et al. Characterization of Classical and Nonclassical Fabry Disease: A Multicenter Study. JASN. 2017;28 (5):1631–1641

ALT: Alanine aminotransferase (ref: <50U/L). CK: Creatine Kinase (ref: 39-308U/L). GI symptoms: Gastrointestinal symptoms

DBS AGAL activity: Dried blood spot α-Gal A activity (ref: 2.40-17.65 µmol/l/h). DBS Lyso-GL-3: Dried blood spot glycosphingolipids (ref: <1.11 ng/ml)

PAB: Prealbumin (ref: 150-300 mg/L). 25(OH)D: 25 hydroxyvitamin D (ref: 35-150nmol/L)

All patients were born full-term with a birth weight > 3 kg except one patient who was preterm at 36 weeks with a birth weight of 2.4 kg. All children except case 6 and 9 had a height in the normal range. Seven of the fourteen children (50%) with the classical phenotype had a BMI below the 15th percentile of the age- and sex-matched reference population. The BC analysis of the 18 children is shown in Table 2.

**Table 2:** Body composition of patients through Whole body dual energy X-ray scans

Case No.	Weight (kg)	Height (m)	BMI (kg/m^2^)	FMI (kg/m^2^)	FFMI (kg/m^2^)	Z-Score	MM (kg)	ASM (kg)	ULSM (kg)	LLSM (kg)	MMI (kg/m^2^)	ASMI (kg/m^2^)	ULSMI (kg/m^2^)	LLSMI (kg/m^2^)
1	40	1.74	13.21	2.82	10.38	-2.1	29.80	13.67	3.23	10.44	9.84	4.52	1.07	3.45
2	63.5	1.64	23.61	9.53	14.04	-0.2	35.70	15.11	2.95	12.16	13.27	5.62	1.10	4.52
3	21.4	1.24	13.92	4.43	9.48	-1.4	13.92	5.57	1.23	4.34	9.05	3.62	0.80	2.82
4	52	1.60	20.31	5.53	14.61	0.1	35.95	15.73	3.60	12.13	14.04	6.14	1.41	4.74
5	36	1.46	16.89	5.86	10.98	-1.1	22.20	9.07	2.30	6.77	10.41	4.26	1.08	3.18
6	37	1.49	16.67	5.81	10.77	-3.7	22.97	8.87	2.22	6.65	10.35	4.00	1.00	3.00
7	25	1.39	12.94	2.81	10.06	-1.7	18.43	7.18	1.67	5.51	9.54	3.72	0.86	2.85
8	32.5	1.51	14.25	3.10	11.11	-0.1	24.09	10.29	2.43	7.86	10.57	4.51	1.07	3.45
9	36	1.61	13.89	3.61	10.25	-1.8	25.36	10.73	2.12	8.61	9.78	4.14	0.82	3.32
10	33	1.45	15.70	4.39	10.85	-0.7	21.77	8.94	2.04	6.90	10.35	4.25	0.97	3.28
11	37	1.50	16.44	4.41	11.93	-1.9	25.57	10.59	2.53	8.06	11.36	4.71	1.12	3.58
12	24.5	1.32	14.06	3.64	10.19	-1.7	16.65	6.44	1.67	4.77	9.56	3.70	0.96	2.74
13	23	1.29	13.82	3.59	9.97	0.3	15.86	6.47	1.60	4.87	9.53	3.89	0.96	2.93
14	27	1.41	13.58	3.79	9.58	0.1	17.95	7.21	1.38	5.83	9.03	3.63	0.69	2.93
15	48	1.57	19.47	6.29	12.97	-1.9	30.70	13.55	3.15	10.4	12.45	5.50	1.28	4.22
16	56	1.56	23.01	10.08	12.54	-2.6	28.64	13.14	3.21	9.93	11.77	5.40	1.32	4.08
17	50	1.81	15.26	2.99	11.63	-2.7	36.14	13.93	3.47	10.46	11.03	4.25	1.06	3.19
18	52	1.48	23.74	11.57	12.08	1.5	24.76	9.52	2.43	7.09	11.30	4.35	1.11	3.24

**Fig. 2:**
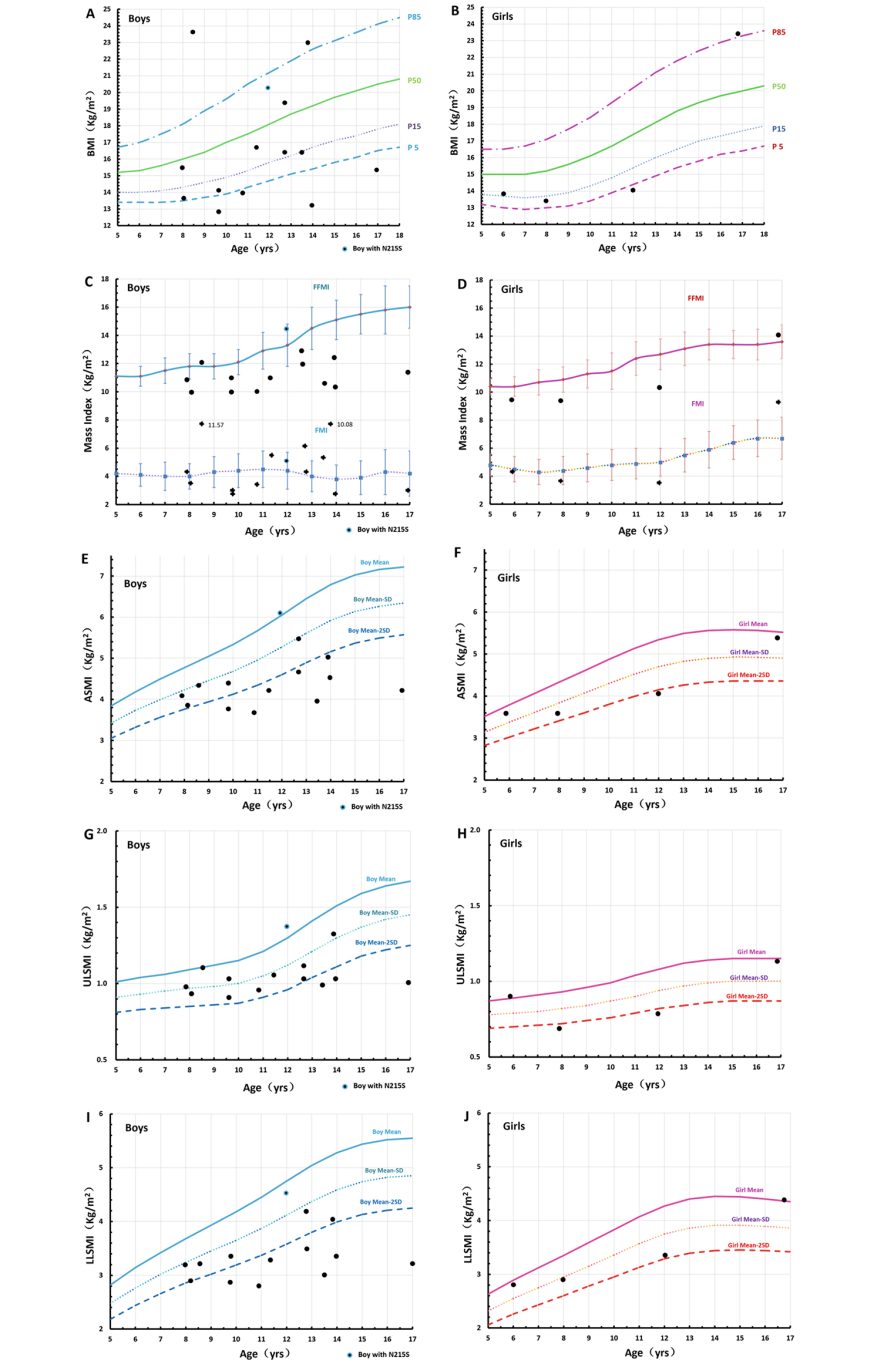
BMI and body composition of FD children applied to reference curves of healthy children in China. All the reference curves of healthy children were adapted from References with permission from the corresponding author professor Jie Mi

DXA was used to understand the physical condition of the children with FD. The data were compared with the normal values of healthy Chinese children and adolescents aged 6–17 years [9, 10]. In boys, the FMI value was below normal in 3 patients, and the FFMI value was abnormal in 12 patients. In the four girls, 1 had an abnormal FMI value and 3 had an abnormal FFMI value. Compared with the normal BC values in Chinese children [9], 8 of the 13 (61.5%) patients with the classical phenotype had a significant reduction in the muscle mass index (MMI), appendicular skeletal muscle mass index (ASMI) and lower limb skeletal muscle mass index (LLSMI) (-2 SD below normal) (Fig. 2). However, in Case 4, the N215S mutation was reported as a late-onset phenotype and appeared to have less of an effect on skeletal muscle.

Compared with the reference values of healthy children, FFMI, MMI, ASMI, ULSMI and LLSMI values were significantly decreased in children with Fabry disease (Table 3). None of the patients had a history of fractures and 4 boys met the diagnosis criteria of low bone mass (areal BMD Z scores of ≤ − 2.0).

**Table 3:** BMI and Body composition analysis of Fabry children (compared with normal reference values for children)

	$\stackrel{-}{X}\pm S$	Minimum	Maximum	Average Z-score (95% confidence interval)	t	P
BMI	16.71 ± 3.71	12.94	23.74	-0.49(-1.55,-0.58)	-0.943	0.359
FMI	5.24 ± 2.63	2.81	11.57	0.80(-0.37,1.96)	1.453	0.164
FFMI	11.30 ± 1.48	9.48	14.61	-1.53(-2.10,-0.95)	-4.827	< 0.001*
MMI	10.70 ± 1.43	9.03	14.04	-1.46(-1.89,-1.03)	-5.957	< 0.001*
ASMI	4.46 ± 0.75	3.62	6.14	-1.52(-2.00,-1.04)	-5.623	< 0.001*
ULSMI	1.04 ± 0.18	0.69	1.41	-1.19(-1.60,-0.78)	-5.048	< 0.001*
LLSMI	3.42 ± 0.60	2.74	4.74	-1.58(-2.09,-1.08)	-5.789	< 0.001*

* P < 0.001

Correlation analysis showed that the ASMI was correlated with age, body weight, BMI and creatinine levels but not with prealbumin, albumin, haemoglobin, blood calcium, alanine aminotransferase, creatine kinase, or 25 hydroxyvitamin D levels (Table 4).

**Table 4:** Pearson correlation coefficients between ASMI and clinical characteristics

	ASMI	
	r	P
Age	0.529*	0.024
Weight	0.82**	0.0001
BMI	0.731**	0.0001
α-Gal A activity	0.059	0.815
Lyso-GL-3	-0.019	0.942
Albumin	-0.143	0.572
PAB	0.462	0.054
hemoglobin	0.207	0.410
Calcium	-0.022	0.932
ALT	-0.236	0.346
CK	0.111	0.661
Cr	0.488*	0.040
25(OH)D	-0.103	0.684

* P < 0.05, ** P < 0.01

Overall, the data revealed that underweight due partly to low skeletal muscle mass, particularly in the lower extremities, was a common early symptom in Fabry patients. This phenomenon is more prominent in children with the classical phenotype.

## Discussion

According to the clinical features and onset, FD is divided into a severe, classical phenotype and a generally milder late onset phenotype [11]. Thirteen boys with the classical phenotype and five children (1 boy with the N215S mutation and 4 girls) with the late-onset phenotype were included in this study. The results suggested that decreased skeletal muscle mass was common in early FD patients, especially those with the classical phenotype.

Low BMI was reported in adult FD patients. A study by Mersebach H revealed that 15% of 53 adult FD patients had a BMI below 20 kg/m^2^ [12]. In this study, no significant difference was found in the BMI of children with FD compared with the reference values of normal children, which may be related to the slow progression of the disease. The BMI of 7 boys (7/13, 53.8%) with classical FD was below the 15th percentile of the age- and sex-matched reference population. Two children (2/18, 11.1%) were short in height, while 8 children (9/18, 50%) were low in weight, which was more obvious in those with the classical phenotype (8/13, 61.5%). This suggested that low BMI was mainly due to underweight, which reminded us to pay attention to nutritional status in these children.

Underweight in FD patients is generally thought to be primarily associated with gastrointestinal involvement [13, 14] which is common but nonspecific and is often overlooked in these patients. Patients may present with paroxysms of pain, diarrhoea, and constipation. The gastrointestinal symptoms of the children in this study did not appear to be significant. The lack of gastrointestinal symptoms in children with FD may be related to their difficulty in describing abdominal discomfort. It is also unlikely to be noticed by parents of children with FD if there are no obvious vomiting or diarrhoea symptoms and routine tests are normal (liver and kidney function, haemoglobin and so on). In addition, glycosphingolipids are gradually deposited in the gastrointestinal tract after birth [15], and sometimes this slow accumulation process makes symptoms difficult to detect. However, all the children had normal levels of prealbumin, albumin and haemoglobin, suggesting that their nutritional status was not poor; therefore, the contribution of other factors needs to be considered.

DXA is the “gold standard” to study BC [16] and it has been widely used in the study of obesity and various diseases affecting bone or muscle mass. To further clarify the cause of underweight, DXA is being used to help us further understand this rare disease. BC has not been studied in children with FD to the best of our knowledge. Basically, normal laboratory test results (normal prealbumin, albumin and haemoglobin levels) suggested that the nutritional status of these children was not very poor. The obvious abnormal FFM and FFMI values caught our attention. Underweight due to the significant reduction in FFMI and mostly normal FMI values for BC could not be fully explained by nutrient intake or absorption disorders. Normal laboratory test results also supported this point. FFM consists mostly of skeletal muscle and bone tissues. By calculating MM, ASM, ULSM, LLSM and related indices, we further determined that underweight in these children was mainly due to low muscle mass. We also noted that two boys (Case 16 and 18) with classical FD had BMIs above the 85th percentile. However, further BC analysis found that this was mainly due to the increase in the FMI value, and the ASMI value was still below normal (mean-1 SD), which further confirmed our findings.

MM accounts for 35–45% of our body weight. Previous literature has reported that skeletal muscle involvement in FD patients manifests as muscle cramps in the early phase and muscle pain, fatigability, and asthenia in later phases, limiting daily activities and reducing quality of life [17, 18]. However, skeletal muscle has rarely been studied previously. The lack of attention may be because skeletal muscle injury is not as life-threatening as heart muscle injury, and the daily activities of these patients are not as severely affected as those with myogenic disorders, which severely affect muscle movement. A previous study showed that myocardial involvement was more serious than skeletal muscle involvement in FD patients [19]. In a study of 12 FD patients, echography showed muscle disarray and hyperechogenic areas in patients older than 35 years, and mild signs of myopathy in electromyography. Muscle cells were significantly less affected than cardiomyocytes [17].In contrast, pathological examination of myofibers in an 18-year-old male FD patient revealed significant lesions and the accumulation of medullary bodies [20].

The causes of low skeletal muscle mass in FD patients may require more research. In addition to the direct destruction of muscle fibres due to abnormal substrate deposition, the abnormal capillary function caused by the destruction of intramuscular vascular endothelial cells may also be one of the reasons. Relevant evidence that GL-3 deposition of intramuscular capillary endothelial cells is shown in the limited literature on muscle involvement in FD patients [24, 25].

Notably, FD is also a risk factor for the occurrence of bone mineral abnormalities. Almost half of the adult FD patients had femoral neck osteopenia. Menopausal women and FD patients with ESRD are more likely to progress to osteoporosis [12]. It is believed that impaired kidney function, underweight, gastrointestinal involvement, physical inactivity, poor absorption of vitamin D, the accumulation of undegraded substrate in bone tissues and other factors lead to osteoporosis. Our results suggested that a reduction in muscle mass may also be involved in the development of osteoporosis in FD patients, since there is solid evidence that low muscle mass is associated with a reduction in bone parameters during growth and increases the risk of osteoporosis in adulthood [26, 27].

In addition, since creatinine is a product of muscle metabolism in the body, low muscle mass can affect blood creatinine levels, which may make clinical assessments of the estimated glomerular filtration rate (eGFR) inaccurate. As glomerular hyperfiltration (eGFR > 135 ml/min/1.73 m^2^) was previously considered to be a relatively common clinical feature in young FD patients [28–30], it may be more reasonable to measure the GFR using nuclear medicine techniques.

In summary, skeletal muscle was significantly affected in paediatric FD patients, and the greatest reduction was in the lower extremities. Its occurrence may be related to the destruction of muscle fibres or intramuscular capillary endothelial cells through the abnormal deposition of glycosphingolipids.

### Limitations

As a rare disease, the sample size of FD patients in the present study was not very large, which may skew the results of the study, and further studies with larger sample sizes are needed. All the children in this study were from China, and there may be ethnic and regional differences.

## Conclusions

This is the first study to examine body composition and muscle mass in early FD patients. We found that skeletal muscle involvement was common in our paediatric patients, suggesting that low skeletal muscle mass may be one of the early manifestations of FD. Physicians need to pay close attention to this aspect in children with FD.

## Electronic supplementary material

Below is the link to the electronic supplementary material.

Supplementary Material 1

## Data Availability

Not applicable.

## Abbreviations

FD: Fabry disease
α-Gal A: α-galactosidase A
GL-3: Globotriaosylceramide
Lyso-GL-3: Glycosphingolipids
BMI: Body mass index
BC: Body composition
DXA: Dual energy X-ray absorptiometry
FM: Fat mass
FMI: Fat mass index
FFM: Fat free mass
FFMI: Fat free mass index
MM: Muscle mass
MMI: Muscle mass index
ASM: Appendicular skeletal muscle mass
ASMI: Appendicular skeletal muscle mass index
ULSM: Upper limb skeletal muscle mass
LLSM: Lower limb skeletal muscle mass
ULSMI: Upper limb skeletal muscle mass index
LLSMI: Lower limb skeletal muscle mass index
